# Intramuscular schwannoma presenting as treatment-resistant lumbar radiculopathy: Case report

**DOI:** 10.1016/j.inpm.2025.100560

**Published:** 2025-03-05

**Authors:** Moshe Spatz, Madison O'Donnell, Amir Gamil, Brett Gerstman

**Affiliations:** a Department of Physical Medicine and Rehabilitation, Rutgers, New Jersey Medical School, Newark, NJ, USA; b New York Institute of Technology College of Osteopathic Medicine, Glen Head, NY, USA; c Rutgers, New Jersey Medical School, Newark, NJ, USA

## Abstract

Intramuscular schwannomas are rare tumors originating from Schwann cells in the peripheral nervous system. These tumors typically occur along small motor nerves deep within the muscle, presenting diagnostic challenges for clinicians due to their rarity and nonspecific symptoms. They can induce pain that is resistant to interventions, and surgical removal is often the only method that provides relief of symptoms. We present a case of a 65-year-old male with low back pain and concurrent right anterior thigh pain and paresthesia. Despite extensive evaluation by numerous specialists and thorough diagnostic testing, including spinal MRIs and electrodiagnostic studies, no clear diagnosis was initially established. His symptoms remained resistant to medical and interventional treatments. Ultimately, MRI imaging of the right thigh revealed a mass measuring approximately 2 cm in the vastus medialis. Following excision of the mass, the patient experienced immediate symptom relief. This case highlights the importance of maintaining a broad differential when evaluating low back pain with associated lower extremity symptoms. We aim to highlight the importance of recognizing this possibility of extra-spinal pain generators.

## 1 Background

Schwannomas are benign, encapsulated tumors that arise from Schwann cells which form the myelin sheath around peripheral nerves. Due to their slow-growing nature, symptoms can be vague, nonspecific, and chronic [1,2]. Nerve sheath tumors classically form along major nerves which may cause symptoms that mimic radiculopathy, such as pain and paresthesia [3,4]. Spinal schwannomas are typically diagnosed as an incidental finding on magnetic resonance imaging (MRI) [4].

Peripheral schwannomas occur less frequently and can be even more difficult to diagnose, as they will not be detected with routine spine MRI imaging. Direct palpation of the mass may elicit tenderness and reproduce symptoms. In some instances, the only clinical sign is insidious pain and swelling, making it difficult to diagnose [1,5,6].

We present a case of a 65-year-old male whose initial symptoms were suggestive of spinal pathology. His prolonged course and lack of response to interventional pain therapies prompted a more comprehensive workup resulting in the discovery of an intramuscular schwannoma.

## 2 Case report

A 65-year-old male presented with right-sided anterior thigh pain with associated axial low back pain. He described the pain as shooting, with a severity rating as high as 9/10. The pain radiated into the anteromedial aspect of his right thigh. The pain occurred constantly and impaired his ability to walk and perform activities of daily living (ADLs) for the previous five months.

Physical exam demonstrated 5/5 motor strength in his bilateral lower extremities. Sensation to pinprick and light touch were intact. Coordination and reflexes were preserved. Femoral abduction and external rotation (FABER), Straight Leg Raise, and Slump tests were negative bilaterally. Heel-to-toe walk tests were performed without difficulty. There was no tenderness to palpation throughout the lower back and right thigh. and soft tissue abnormalities were observed.

Past medical history was remarkable for hypertension and hyperlipidemia, with no history of diabetes mellitus. Social history was notable for light tobacco use (less than 10 cigarettes per day) and occasional alcohol intake. Family history was notable for his father, who developed prostate cancer. He had previously tried conservative treatments, including physical therapy, oral corticosteroids, and NSAIDs, without meaningful relief.

An MRI of his lumbar spine was ordered and revealed L4-L5 moderate-severe neural foraminal stenosis. Electrodiagnostic testing findings were suggestive of L4 and L5 radiculopathy, with no evidence of peripheral polyneuropathy. Given these findings, a right L4-L5 transforaminal epidural steroid injection was performed. Immediately following the injection, the patient reported 0 % relief of his symptoms. Two months later, an additional attempt of a right, transforaminal, epidural corticosteroid injection was performed at L3, with no relief of symptoms. Pregabalin was trialed and titrated to increasing dosages with no reported changes. The patient concurrently obtained evaluations with multiple neurologists and spinal surgeons at academic institutions without a clear diagnosis or treatment plan.

During a follow-up visit five months after the initial presentation, the patient reported worsening dysesthesias in the right thigh. On exam, severe point tenderness was noted over the right anterior thigh. A new weakness (4/5 motor strength) was noted in knee extension on the right. Sensation to pinprick and light touch was decreased along the dermatomes of L4, L5, and S1.

**Fig. 1:**
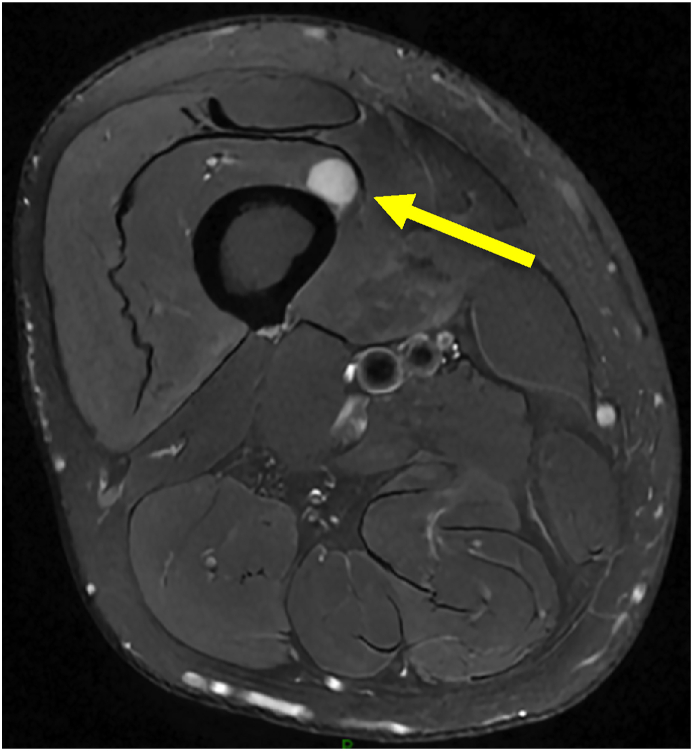
MRI of the lower extremity showing a 1.2 cm schwannoma in the vastus intermedius.

These findings led to increased suspicion of an intrinsic pathology within the right thigh as a pain generator. An MRI of the right femur was ordered and revealed a 2 cm soft tissue mass within the vastus intermedius muscle (see Fig. 1), and he was subsequently referred to an orthopedic oncologist for further management. Surgery revealed a 2 cm mass deep to the interval between the vastus medialis and rectus femoris. The mass was enveloped by the overlying fascia and an adjacent nerve was observed to be exiting the mass. The sheath of the nerve was opened longitudinally, and the mass was carefully enucleated from the sheath while leaving nerve fascicles intact. A specimen was obtained and a biopsy was performed which confirmed the mass was a schwannoma. Following the procedure, the patient reported complete relief of his symptoms.

## 3 Discussion

Schwannomas have a prevalence of 5% among soft tissue neoplasms, and most commonly occur between the second and fifth decade of life [4,7,8]. Malignancy is rare unless the tumor is associated with Type 2 Neurofibromatosis [9]. Schwannomas commonly develop along the spine (49%), and less frequently in the lower extremities [10].

Intraspinal schwannomas are frequently detected as an incidental finding with routine spine imaging. They classically demonstrate a split-fat sign, low signal margin, and a hyperintense rim [11,12]. They have a good prognosis, and extraction typically provides complete resolution of symptoms [3]. Spinal schwannomas have a recurrence of 4–6%, with known risk factors including subtotal resection, multilevel involvement, large tumor size, and malignant histopathology [13].

Peripheral schwannomas can occur anywhere along the nervous system [13]. In the lower extremities, they are unlikely to be detected with routine imaging and often go undetected for prolonged periods. The incidence of intramuscular schwannoma is extremely low, and there are no reported studies on their rate of recurrence [14].

Intramuscular schwannomas may have clinical signs such as local pain and swelling, but often symptoms are nonspecific [1,5,6]. They rarely cause motor weaknesses, as they typically arise from small motor nerve branches within the muscle [12]. A paucity of characteristic clinical signs makes intramuscular schwannoma a challenging diagnosis [15].

Our reported patient, whose initial symptoms were right anterior thigh pain with associated low back pain, suggested that his pain was of spinal origin. A comprehensive physical exam did not reveal any signs indicating peripheral pathology. His MRI and electrodiagnostic findings of lumbar radiculopathy initially supported this suspicion, and therapies were targeted to address his L4-L5 spondylolisthesis. It was later determined that the electrodiagnostic and imaging findings were secondary to unrelated spinal pathology. This case is an atypical presentation of symptoms for an intramuscular schwannoma. It is extremely rare for peripheral nerve lesions to present as back pain or radiculopathy, though a few cases of intramuscular schwannomas with similar radicular symptoms have been reported [16,17]. It is possible that this observed phenomenon may be caused by proximal spread along the nerve to the nerve root [15].

Frequent follow-up with the patient over time revealed clues suggestive of a peripheral issue. The schwannoma's location within the vastus intermedius led to entrapment of the femoral nerve, resulting in weakness with knee extension. The development of weakness with tenderness to deep palpation of the anterior thigh suggested that this was an intrinsic leg pathology, and less likely due to L4 radiculopathy. His poor response to epidural corticosteroid injections and neuropathic medications reinforced this suspicion. An MRI was then ordered and demonstrated the 1.2 cm benign schwannoma in the vastus medialis. Full symptomatic relief with surgical excision confirmed that the schwannoma was his primary pain generator. The surgical report described the location of the mass in the middle 1/3rd of the femoral shaft, directly deep to the interval between the vastus medialis and rectus femoris. The adjacent femoral nerve, or a branch of the femoral nerve, was the presumed origin. The patient's low back pain remained unchanged following the surgery and was likely a sequela of his underlying L4-L5 foraminal stenosis and an L5-S1 disc bulge. This was also the most likely explanation of his diminished sensation in the L5 and S1 dermatomes.

A broad differential and consideration of extraspinal etiologies, combined with close follow-up and a comprehensive physical exam at each visit, allowed for an accurate diagnosis and immediate resolution of the patient's symptoms with surgical excision.

## 4 Conclusion

Intramuscular schwannomas are rare, but may present with peripheral pain, and in some instances low back pain with radicular symptoms. As it can be easily detected with appropriate imaging and surgical excision often results in complete resolution of symptoms, it is important for clinicians to consider intramuscular schwannoma in their evaluation. Maintaining a broad differential to rule out less common, but treatable pathologies, can help avoid unnecessary and ineffective interventions.

## Consent

Patient's consent was obtained to disclose case details.

## Declaration of competing interest

The authors declare that they have no known competing financial interests or personal relationships that could have appeared to influence the work reported in this paper.

## References

[1] Moond V., Diwaker P., Golamari R., Jain R. (2021). Intramuscular ancient schwannoma of the axillary nerve. BMJ Case Rep.

[2] Chijiiwa Y., Sano J., Okamura K., Nishio J. (2024). Intramuscular hybrid nerve sheath tumor of the thigh: case report and literature review. In Vivo.

[3] Rustagi T., Badve S., Parekh A.N. (2012). Sciatica from a foraminal lumbar root schwannoma: case report and review of literature. Case Rep Orthop.

[4] Hyseni F., Harizi E., Blanco R. (2022). Lumbar radiculopathy associated radicular schwannoma: a case report and literature review. Radiol Case Rep.

[5] Koike N., Hasegawa H., Matsuzaki H., Oshima T. (2022). Posterior cervical intramuscular schwannoma within the trapezius muscle: a case report. Turk Arch Otolaryngol.

[6] Pham Quang V., Hoang Quoc H., Nguyen B., Ngo Quang C., Nguyen Chi H., Nguyen N. (2023). Giant schwannoma on the lower leg: a case report and review of the literature. Int J Surg Case Rep.

[7] Addi Palle L.R., Depa V.G.R., Shah K., Soto C.J., Aychilluhim B.A., Rakhunde V.V. (2023). Peripheral schwannoma presenting as a retro-malleolar mass: a case report. Cureus.

[8] Kransdorf M.J. (1995). Benign soft-tissue tumors in a large referral population: distribution of specific diagnoses by age, sex, and location. Am J Roentgenol.

[9] Kim D.H., Murovic J.A., Tiel R.L., Moes G., Kline D.G. (2005). A series of 397 peripheral neural sheath tumors: 30-Year experience at Louisiana State University Health Sciences Center. J Neurosurg.

[10] Zipfel J., Al-Hariri M., Gugel I. (2021). Surgical management of sporadic peripheral nerve schwannomas in adults: indications and outcome in a single center cohort. Cancers.

[11] Lee S.K., Kim J.Y., Lee Y.S., Jeong H.S. (2020). Intramuscular peripheral nerve sheath tumors: schwannoma, ancient schwannoma, and neurofibroma. Skelet Radiol.

[12] Salunke A.A., Chen Y., Tan J.H. (2015). Intramuscular schwannoma: clinical and magnetic resonance imaging features. Singap Med J.

[13] Shimose S., Sugita T., Kubo T. (2007). Major-nerve schwannomas versus intramuscular schwannomas.

[14] Conti P., Pansini G., Mouchaty H., Capuano C., Conti R. (2004). Spinal neurinomas: retrospective analysis and long-term outcome of 179 consecutively operated cases and review of the literature. Surg Neurol.

[15] Aktas I., Palamar D., Akgun K. (2015). Lateral pectoral nerve injury mimicking cervical radiculopathy. Clin J Sport Med.

[16] D'Silva K.J., Dwivedi A.J., Barnwell J.M. (2003). Schwannoma of the psoas major muscle presenting with abdominal and back pain. Dig Dis Sci.

[17] Agarwal D.K., Thamatapu E., Sanyal S., Krishnan P., Encountered Rarely, Considered Seldom (2018). Posterior tibial nerve schwannoma mimicking lumbar radiculopathy. J Neurosci Rural Pract.

